# Recovery of Meteorological Data for the Observatory of A Guarda, Spain

**DOI:** 10.1371/journal.pone.0039281

**Published:** 2012-06-29

**Authors:** Juan A. Añel, Marcos Blanco-Durán, Luis Gimeno, Laura de la Torre

**Affiliations:** 1 Smith School of Enterprise and the Environment, University of Oxford, Oxford, United Kingdom; 2 EPhysLab, Facultade de Ciencias, Universidad de Vigo, Ourense, Galicia, Spain; Ohio State University, United States of America

## Abstract

We herein describe the recovery of a series of data on temperature, humidity, precipitation, evaporation, wind, and local weather conditions from documentary sources obtained from the Jesuit observatory of A Guarda (Galicia, Spain) for the period 1881–1896. The data were digitized and made available in accessible electronic formats. Comparisons were made with present-day meteorological data obtained from two nearby stations. We further believe that the discovery of some new complementary documentary sources made during the present research could be a basis for future data recovery efforts. Among these new results, early ozone data from the period are of outstanding importance to meteorologists.

## Introduction

The recovery of instrumental meteorological records from printed manuscripts are of great importance for paleo-climatological studies [1]. The International Environmental Data Rescue Organization (IEDRO) states that the main benefits of data recovery lie in the study of climate change, as well as for disease prevention, flood forecasting, the design of safer infraestructure, the prevention of famine and for the general understanding of climate in historical terms. According to the World Meteorological Organization (WMO), such recovery includes the storage of the recovered data in the form of digital media that are easy to read and access [2]. The WMO recently introduced the Mediterranean climate data rescue (MEDARE) initiative, with the aim of developing and improving data recovery activities for the Greater Mediterranean Region [3]. They have been launched in the last years the “Data Rescue at Home” (http://www.data-rescue-at-home.org/) project as an “attempt to digitize historical weather data from all over the globe and the “International Atmospheric Circulation Reconstructions over the Earth (ACRE)” initiative [4]. Instrumental data recovery is of great importance for years before 1940’s because of the lack of good meteorological records, and some efforts have already been made in respect of the North Atlantic region, for example, to reconstruct time series pertaining to the North Atlantic Oscillation (NAO) [5], which is the dominant mode of variability of winter climate in the North Atlantic region [6], [7]. Particularly important in studies of this type are the Spanish historical archives, which are recognized to be a vast store of meteorological information that deserve special attention and could be used as a basis for data recovery efforts [8], [9].

The Society of Jesus (the “Jesuits”) has maintained an interest in the natural sciences since its foundation in 1540. The results of such a scientific tradition (documentary sources, observatories, reports, etc.) are of broad interest in several disciplines, with meteorological and climatological studies being among the most prominent. The Jesuits were a highly educated community and the reliability of their data is likely to be high [10], [11].

Combining these two issues of supply and demand, we herein present the results of a study of recovery and digitization of documentary sources of meteorological data obtained from the Jesuit observatory of A Guarda, Spain. The study was undertaken with the aim of contributing to global efforts in data rescue and for the study of regional climatological patterns for the northwestern Iberian Peninsula, a region strongly affected by the NAO [12], [13]. The national weather services of Spain have previously recovered and digitized large amounts of meteorological data [14]. However, the location and period of measurement of the data here presented herein, together with the fact that the data were only ever sporadically transmited to the national central observatory of Spain [15], make their systematic recovery particularly valuable.

The remainder of the paper is organized as follows: we first present a materials and methods section, containing information about the observatory, a description of the data series used for comparative purposes, and a description of the existing documentary sources, summarizing their information in a set of tables. Secondly, we present the results of the digitization of the data, some figures in which the historical data are compared with data from the present day, and we also describe a new documentary source discovered during the present study. In the conclusions, we discuss the outcomes of the project and suggest future avenues of enquiry.

## Materials and Methods

### The Observatory of A Guarda and Metadata

The observatory at the centre of our study was located in A Pasaxe, Santa Isabel de Camposancos, A Guarda, Pontevedra (Spain). According to the bibliographic data [16], it was situated at latitude 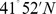 and longitude 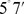 West of Madrid (Spain) (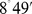 West of the Greenwich meridian). It was part of the “Apóstol Santiago College” and owned by the Jesuits. The college was originally founded in 1872 in the city of A Coruña (approximately two hundred kilometers to the north) with the aim of eventually being the first private university in Spain, and it then moved to A Pasaxe in 1876. In its new location the observatory was located just a few meters from the Miño riverside, which is the geopolitical frontier between Spain and Portugal, and just a few kilometers from the Atlantic Ocean [11], [15].

**Figure 1:**
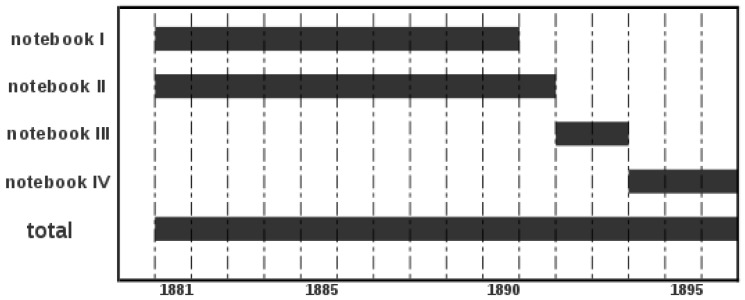
Period covered by the data recovered.

The observatory was at sea level (though in parts of the documentation is indicated to be in a height of eight meters) in a building with a hexagonal floor in the gardens of the main college. Its height was 2.5 m from floor to ceiling, with a total roof height of 0.8 m. Wooden planks were set in the eatern, southern and western walls like blinds, to avoid the direct impact of solar radiation on the instruments. All the instruments were located at an elevation of 1.5 m above the floor and the altitude of the barometer relative to mean sea level was 8 meters. The instrumentation in the observatory included:

- Walferdin maximum thermometer
- Rutherford minimum thermometer
- Bellani thermograph
- psychrometer
- Tonnelot rain gauge
- Eon Fils barometer
- evaporimeter
- minimum thermometer exposed to solar radiation
- maximum thermometer exposed to solar radiation
- weather vane

The observatory was operational by the end of November of 1880. From 1883 onwards, daily reports of the observations were sent to the Instituto Central Meteorológico de Madrid (the principal meteorological institute of Spain) and the data were published in annual bulletins. Futhermore, between 1881 and 1897 the observatory of A Guarda published a number of bulletins entitled “Colegio de La Guardia. Boletines meteorológicos” (A Guarda College. Meteorological bulletins). At some point between 1903 and 1911, the observatory was moved to a tower builded on the southern side of the college. We found no reference pertaining to the location of the observatory between 1911 and 1917. The observatory stopped working by 1917.

**Figure 2:**
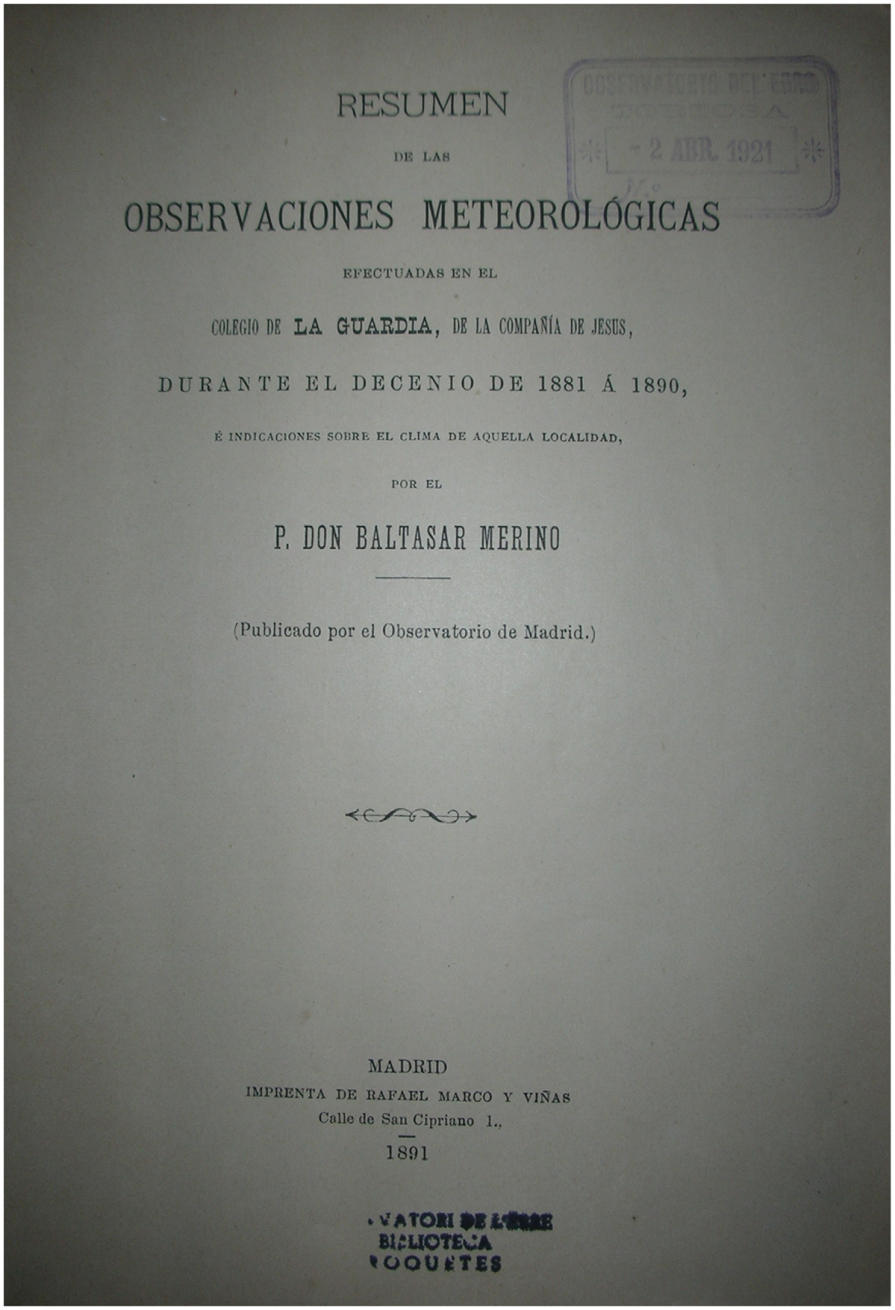
First page of the Notebook I [**16**].

**Figure 3:**
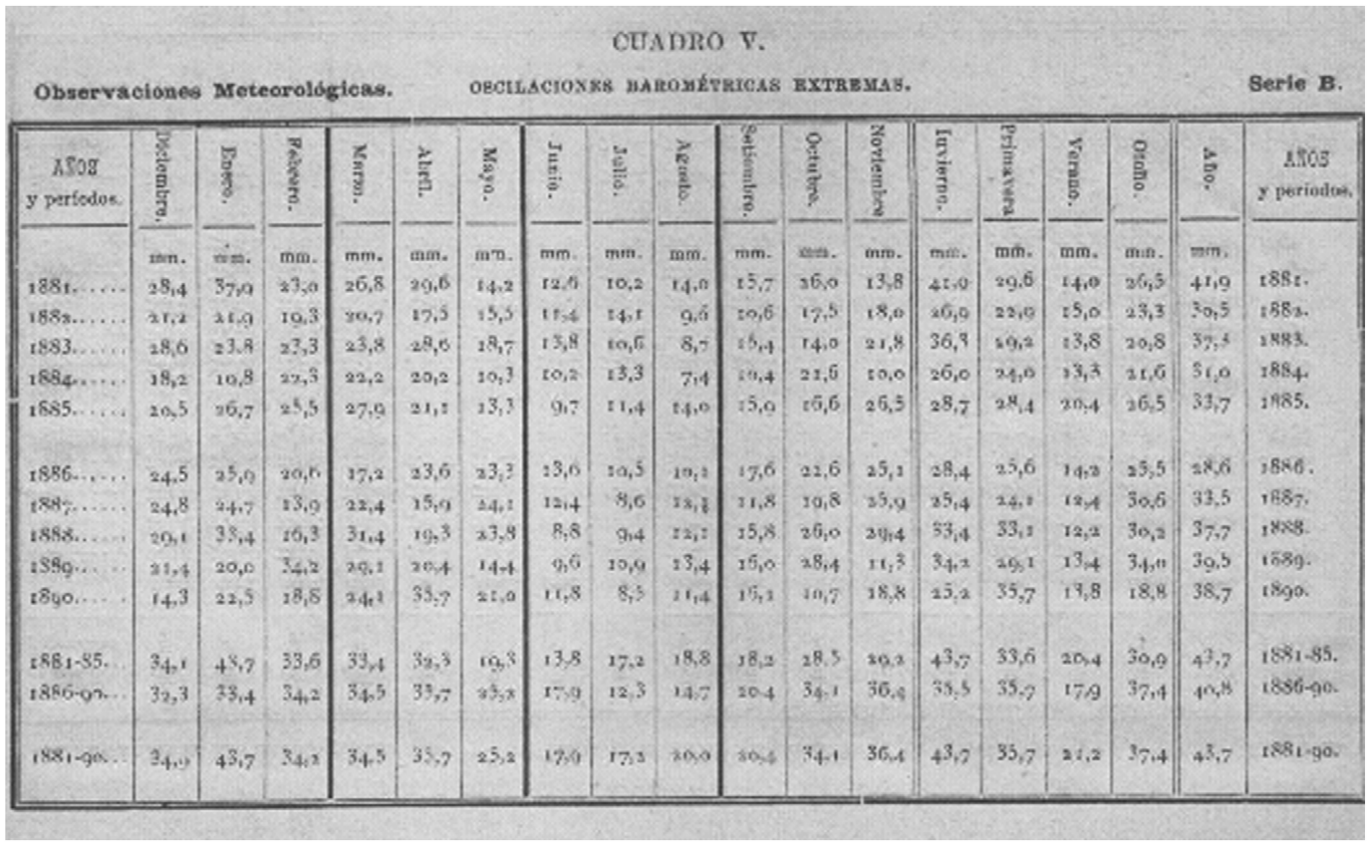
Example of a table showing data on extreme barometric oscillations from Notebook I [**16**].

**Figure 4:**
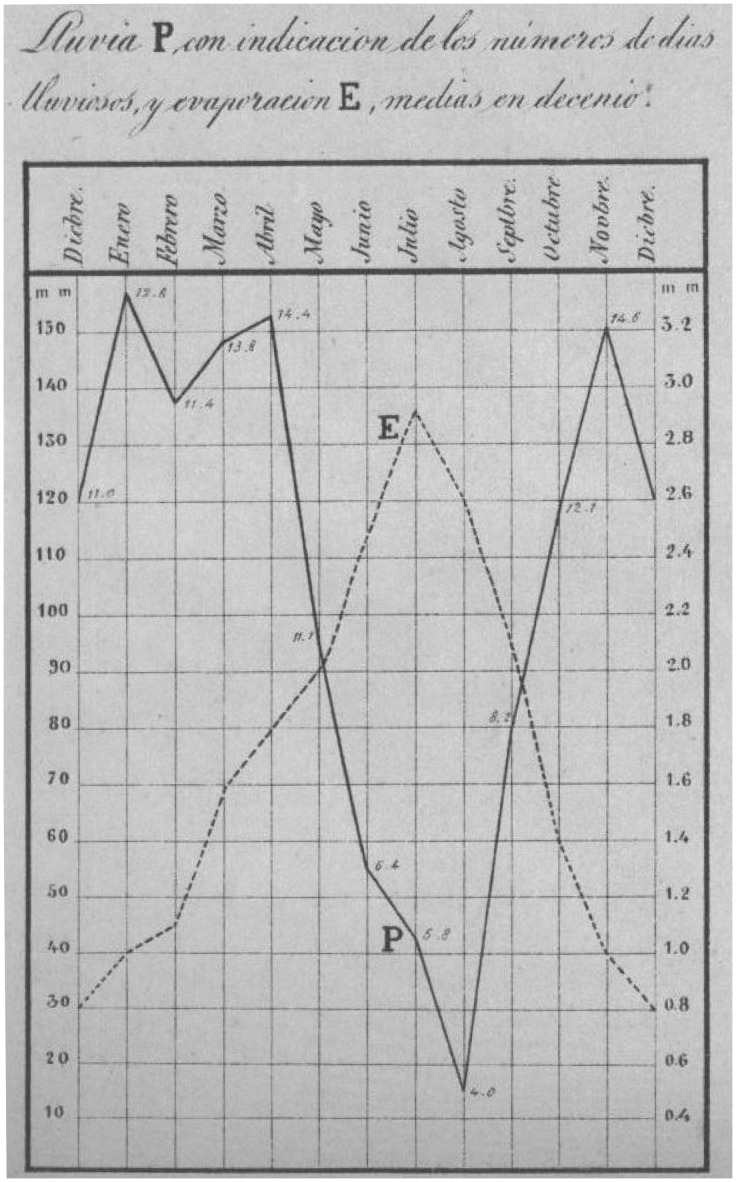
Example of a graph of precipitation and evaporation from Notebook I [**16**].

### Monte Aloia and Vigo-Peinador

In order to compare the data obtained from A Guarda with data from the present day, we used data from the automatic weather station “Monte Aloia” and the meteorological observatory “Peinador”. Monte Aloia is the closer weather station to A Guarda but it lies in a much higher altitude and its data series is short which makes harder the comparison of results based on pressure. Peinador is the closer observatory with a 30 years climatological series.

The Monte Aloia automatic weather station is operated and mantained by the Regional Government of Galicia. We chose this station for three main reasons. Firstly, it lies close to the former observatory of A Guarda, at approximatly 27 km from its original location; secondly, the data series for this station for the period 2001–2010 show a good temporal homogeneity. Moreover, and thirdly, both the data and metadata are publicly available at http://www.meteogalicia.es. The station is located at 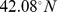 and 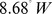, and it lies at an elevation of 484 m above sea level.

**Table 1:** Characteristics of the data contained in the notebooks.

	Formal	Source format	photocopy
		Information type	numeric, text, graphical
		Information format	tables, text
Notebook I		Typing	printed
		Legibility	clear
	Informational	Data coverage	Observatory of A Guarda, 1881–1890
		Quality	good
		Redundancy	reports from the local newspaper “La Integridad”
	Formal	Source format	photocopy
		Information type	numeric, text
Notebook II		Information format	tables, graphs, text
		Typing	printed
		Legibility	clear
	Informational	Data coverage	Observatory of A Guarda, 1881–1891
		Quality	good
		Redundancy	reports from the local newspaper “La Integridad”
	Formal	Source format	photocopy
		Information type	numeric, text
Notebook III		Information format	tables, text
		Typing	printed
		Legibility	clear
	Informational	Data coverage	Observatory of A Guarda, 1892–1893
		Quality	good
		Redundancy	reports from the local newspaper “La Integridad”
	Formal	Source format	photocopy
		Information type	numeric, text, graphical
Notebook IV		Information format	tables, text
		Typing	printed
		Legibility	clear
	Informational	Data coverage	Observatory of A Guarda, 1891–1896
		Quality	good
		Redundancy	reports from the local newspaper “La Integridad”

The observatory located in Peinador (known as Vigo-Peinador) is operated by the Spanish meteorological agency (AEMET). The observatory is located at 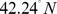 and 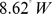, and it lies at an elevation of 261 m above sea level. The distance to the observatory of A Guarda is approximately 45 km. The data and metadata are available in the web page of AEMET (http://www.aemet.es) and at http://eca.knmi.nl provided by the ECA&D project [17].

### Materials

This study is based on the publications and observations made at the observatory by Father Baltasar Merino. Copies of his four notebooks (in Spanish), are kept at the library of the Observatori de l’Ebre, an observatory situated in Roquetes (Spain), which was also originally founded by the Jesuits. Figure 1 shows the period covered by the data recovered for the present study.

**Figure 5:**
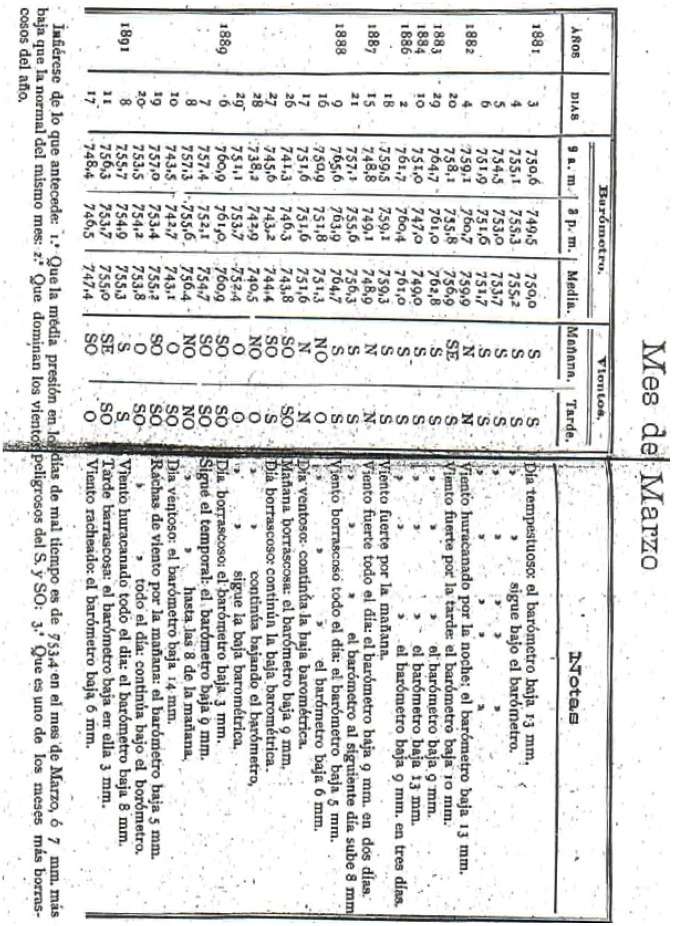
Example of a typical page containing data from Notebook II [**18**].

We now describe the documentary sources by applying the structure recently suggested by Brönnimann et al. [1].

#### Notebook I

Published in 1891, this is the first of the books on the meteorological characterization of the site written by Father Baltasar Merino. Its original title was “Resumen de las Observaciones Meteorológicas efectuadas en el Colegio de La Guardia, de la Compañía de Jesús, durante el decenio de 1881 a 1890” (Summary of the meteorological observations for the A Guarda College, of the Society of Jesus, for the decade 1881 to 1890) [16]. This notebook contains descriptions of the observatory, its location, the materials used to build it, as well as the instrumentation and the metadata for the measurement procedures.

**Figure 6:**
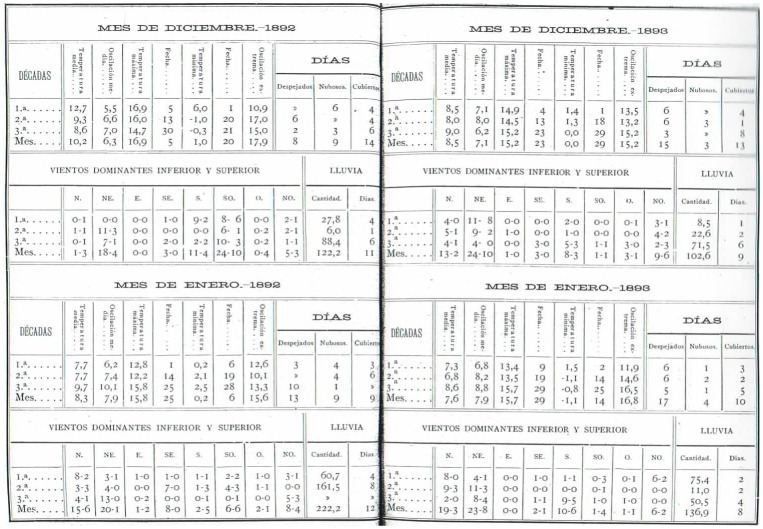
Example of a table showing data split into periods of ten days of dominant winds and precipitation from Notebook III [**19**].

**Figure 7:**
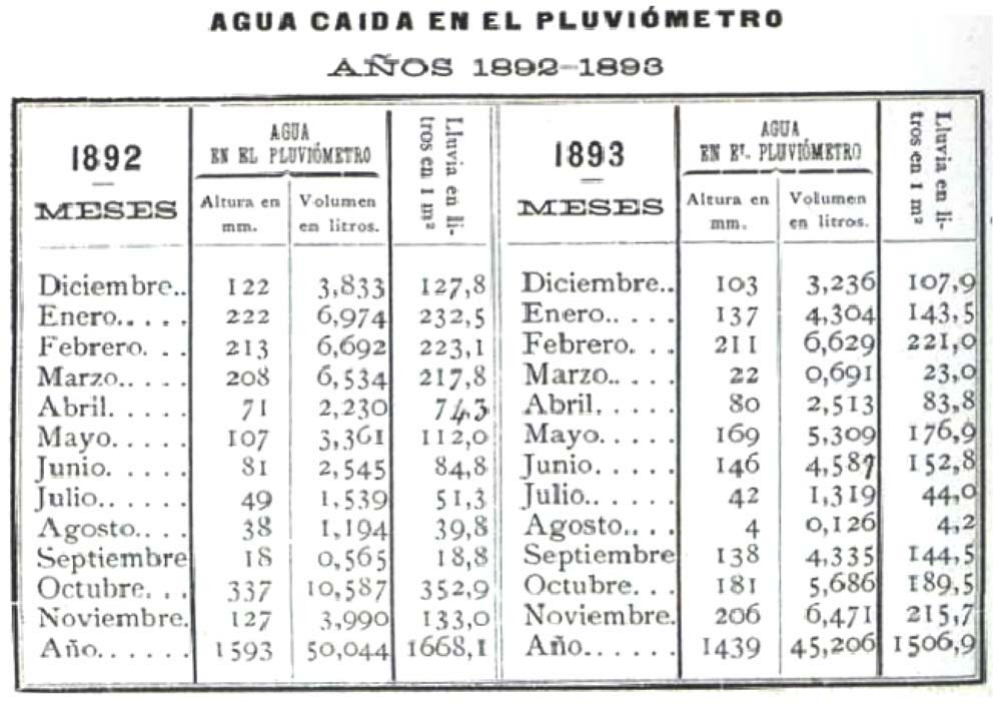
Example of a table showing data on precipitation measured by rain gauge from Notebook III [**19**].

**Figure 8:**
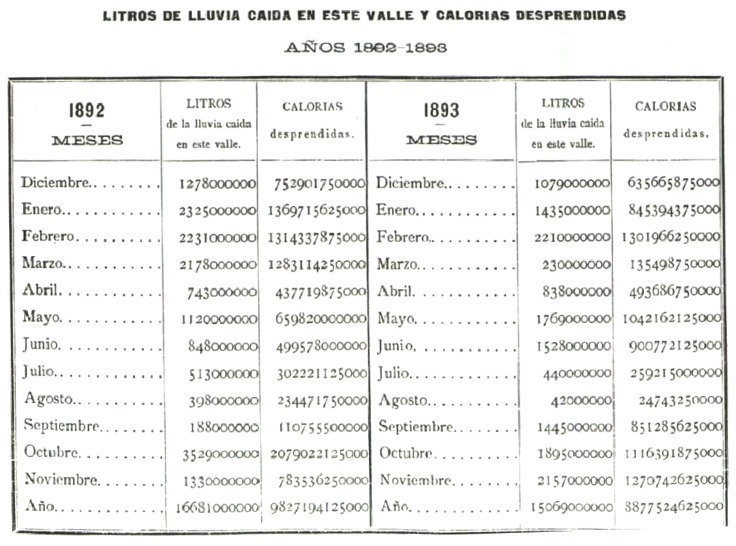
Estimated precipitation for the valley and calories emitted, from Notebook III [**1**].

Figures 2, 3 and 4 show the cover of the notebook and some examples of data included therein. The characteristics of the data contained in the notebook are given in Table 1. The data include readings of pressure and temperature (mean, minimum and maximum), as well as oscillations (diurnal and maximum), mean relative humidity, vapor pressure, precipitation (number of days with rainfall and depth of rainfall), mean evaporation, general observations of weather, as well as wind speed and direction. All the data are shown as monthly, annual and five-year values. The data series are splitted into two different series, namely “A”, where the different variables are displayed jointly in the same table in annual and seasonal periods, and “B”, with a different table for each variable shown (Figure 3). The last part of the notebook contains comparative graphs of the different variables studied. An example is provided in Figure 4.

**Figure 9:**
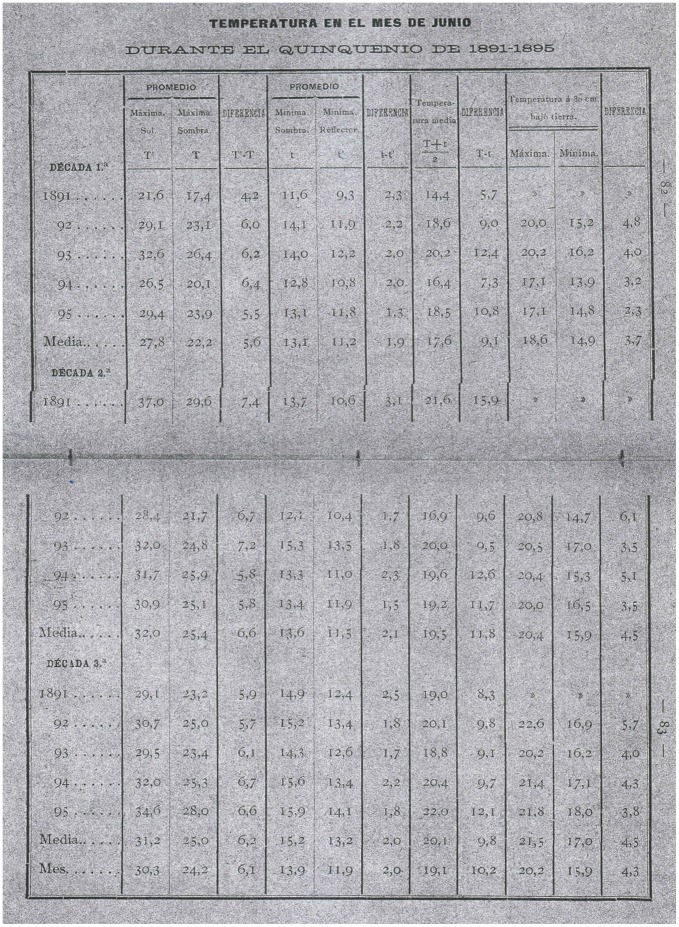
Example of a table showing data from Notebook IV [**20**].

#### Notebook II

Notebook II was published in 1893 under its original title “Estudio sobre las Borrascas en la costa occidental de Galicia” (Study of extratropical cyclones for the western coast of Galicia) [18]. It should be noted that the criterion used in this notebook to denote an extratropical cyclone is not the same as the WMO criterion, as will be explained later. In this notebook Father Merino proposed, at the end of the 19th century, the constitution of a European meteorological service that would focus on the Atlantic region. Table 1 shows the characteristics of the data contained in this notebook, which cover the period 1881–91.

**Figure 10:**
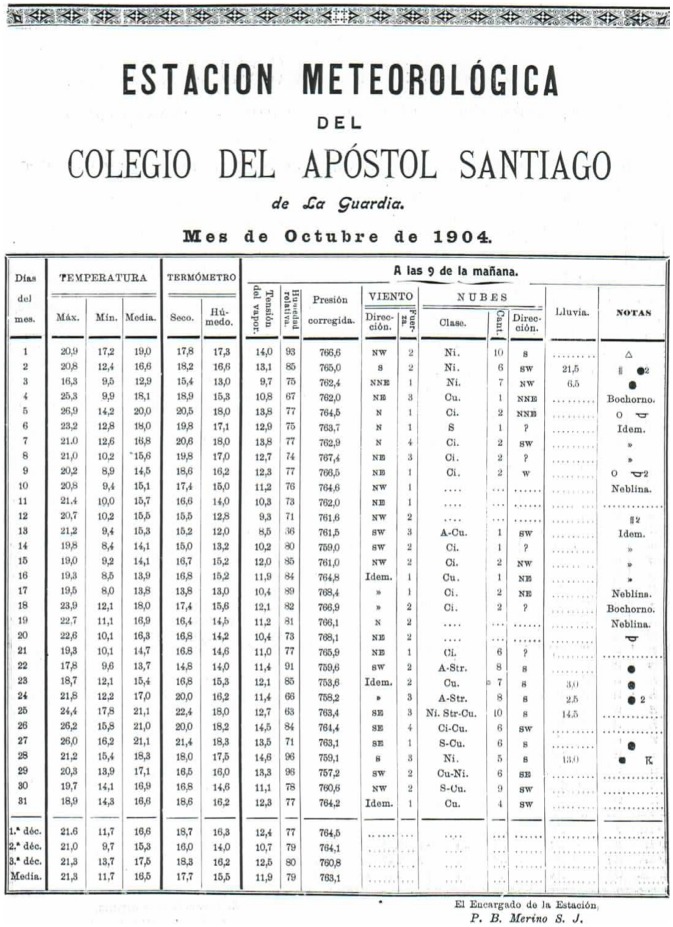
Example of data contained in the reports of Father Orcolaga.

**Table 2:** Characteristics of the data contained in the reports of Father Orcolaga.

Formal	Source format	photocopy
	Information type	numeric, text
	Information format	tables, text
	Typing	printed
	Legibility	clear
Informational	Data coverage	Observatory of A Guarda, 1904–1906
	Quality	good

The main focus of Notebook II is the study of low pressures and of meteorological phenomena that affect sailing. It therefore contains a complete description of the aneroid barometer and its applications. It starts with an analysis of the dangers of low-pressure areas to sailors and contains a complete study of the their characteristics when arriving at the west coast of Galicia. A methodology for forecasting this phenomenon is also proposed. The data contained in the notebook are values of pressure and wind speed and direction for some days, measured twice a day (morning and evening), before, during and after the occurrence of different low pressure systems. Figure 5 shows an example of a typical entry in this book.

**Figure 11:**
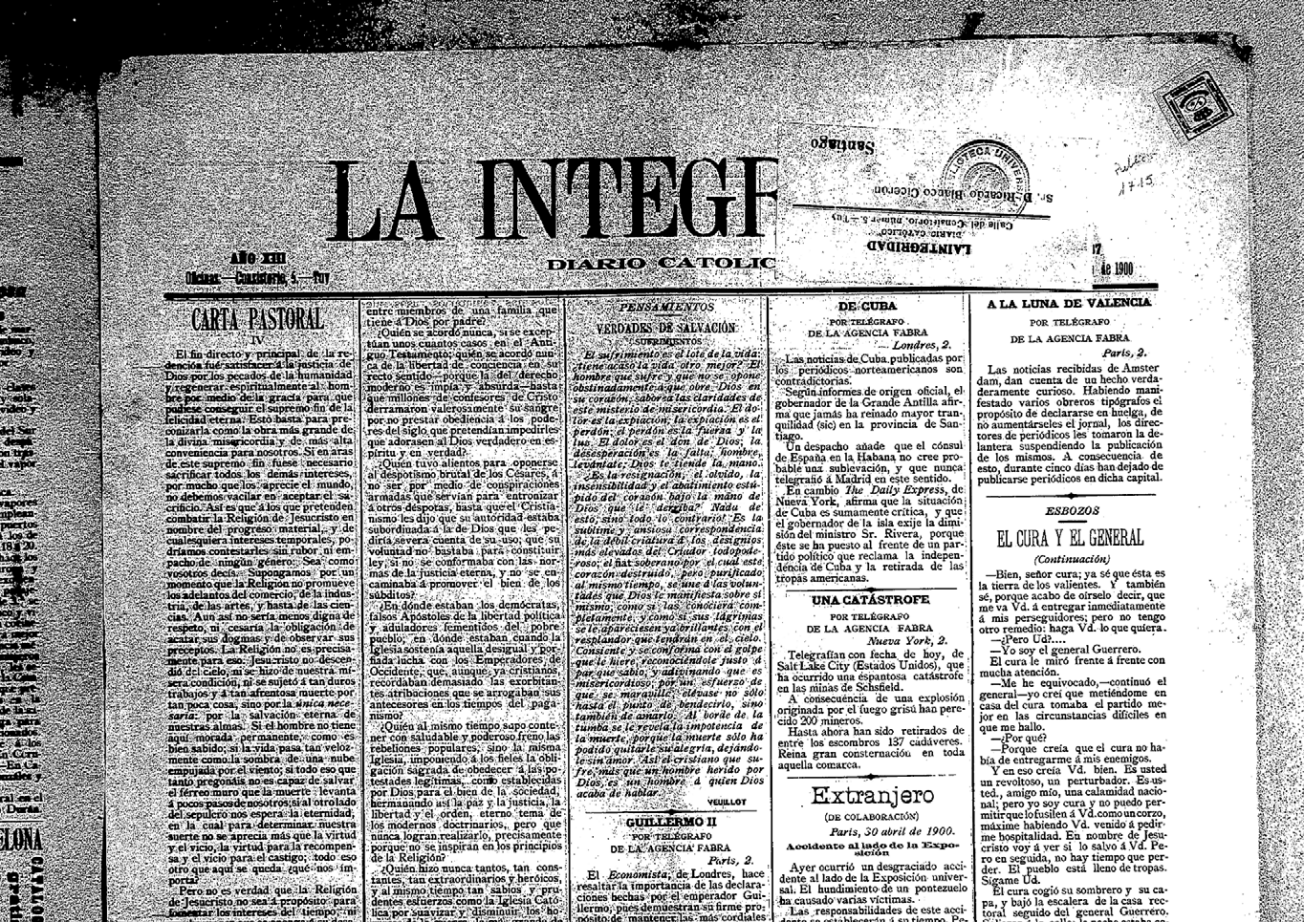
Example of front page of “La Integridad”.

**Figure 12:**
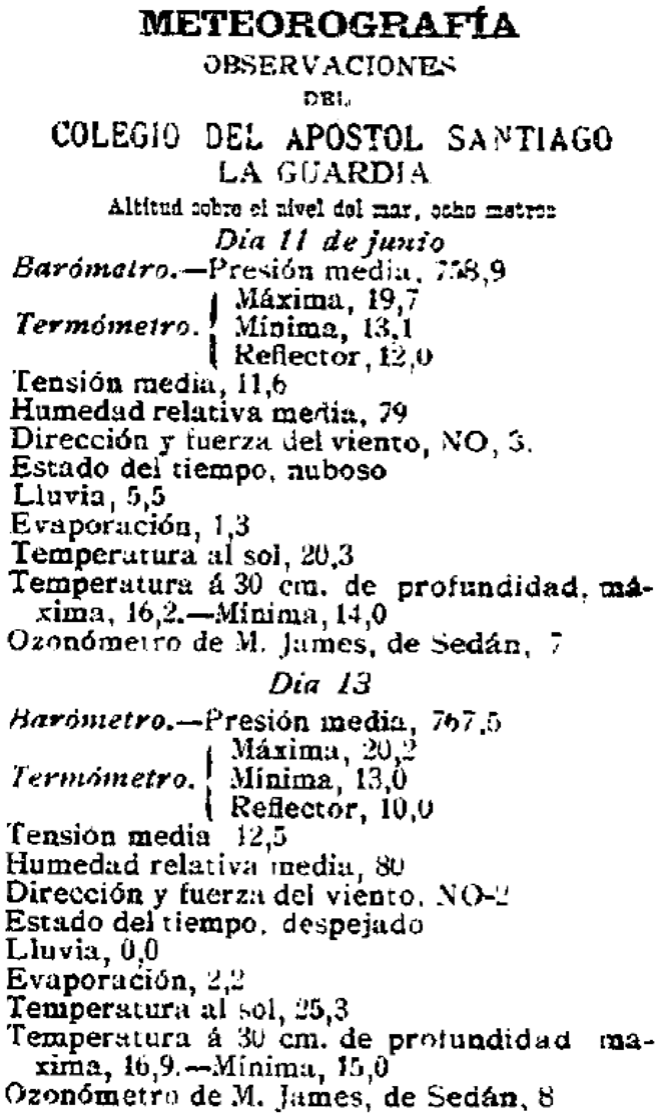
Example of a meteorological report published in “La Integridad”.

**Table 3:** Characteristics of the data contained in La Integridad.

Formal	Source format	microfilm
	Information type	numeric, text
	Information format	tables, text
	Typing	printed
	Legibility	clear
Informational	Data coverage	Observatory of A Guarda
	Quality	good

#### Notebook III

Published in 1894, this notebook was entitled “Observatorio Meteorológico del Colegio de la Compañía de Jesús en La Guardia. Cuaderno Tercero” (Meteorological observatory of the College of the Society of Jesus in La Guardia. Third notebook) [19]. It contains a study of meteoric water and its impact on temperature at A Guarda. It is followed by a complete description of the sources of meteoric water and the process of evaporation in the Atlantic coast of Galicia, with information about its mean temperature, surface and mean daily evaporation. Father Merino explained the process of evaporation for the Atlantic Ocean, focusing on the warmest part of the northern Atlantic Ocean, and estimating an area of 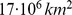 and a sea surface temperature of 25°C. He calculated the rate of evaporation per square metre by taking into account the area under consideration and multiplying it by the mean daily evaporation, and then explained the process from evaporation to precipitation.

The characteristics of the data contained in the notebook are shown in Table 1. The recorded data cover the period 1892–3, and are shown by splitting each month into three periods of ten days (termed “décadas” in the notebook) and comprise:

- mean, maximum and minimum values of temperature
- monthly mean values of temperature
- number of cloudy days and quantification of cloud cover (cloud-free, cloudy, obscured)
- dominant winds

days with precipitation and depth of rainfall (in mm, L and L/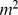)

Figure 6 shows a page of this data as recorded in the notebook. The notebook also contains two tables: the first shows the monthly amount of water recorded in the rain gauge, including the depth in mm, the volume in litres and the corresponding number of litres per square metre (see Figure 7); the second shows an estimate of the total quantity of water (in litres) precipitated in the valley in which the observatory was located (with an estimated total surface area of 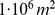 and the corresponding values of calories emitted) (see Figure 8). A procedure for the analysis of dissolved chlorine and ammonia in the rain measured for 1893 and 1894 is also explained.

#### Notebook IV

Published in 1897, the fourth notebook is entitled: “Observatorio Meteorológico del Colegio de la Compañía de Jesús en La Guardia. Cuaderno Cuarto” (Meteorological observatory of the College of the Society of Jesus in La Guardia. Fourth notebook) [20]. It includes meteorological observations for the period 1891–6. The characteristics of this notebook as a source of data are shown in Table 1 and an example of the information that it contains can be seen in Figure 9.

The first part of this notebook contains a study of the relationship between the vegetation and temperature in the drainage basin of the Miño river. Monthly values of temperature are given for the period 1891–6, and the months are split into periods of ten days called “décadas”. For these periods the variables recorded and averaged are the maximum temperature in the sun, the maximum temperature in the shade, and their difference, the minimum temperature in the shade, the minimum reflected temperature and the difference between them, and the mean, maximum and minimum soil temperature at a depth of 30 cm. The flora in the area around the observatory are also described.

**Figure 13:**
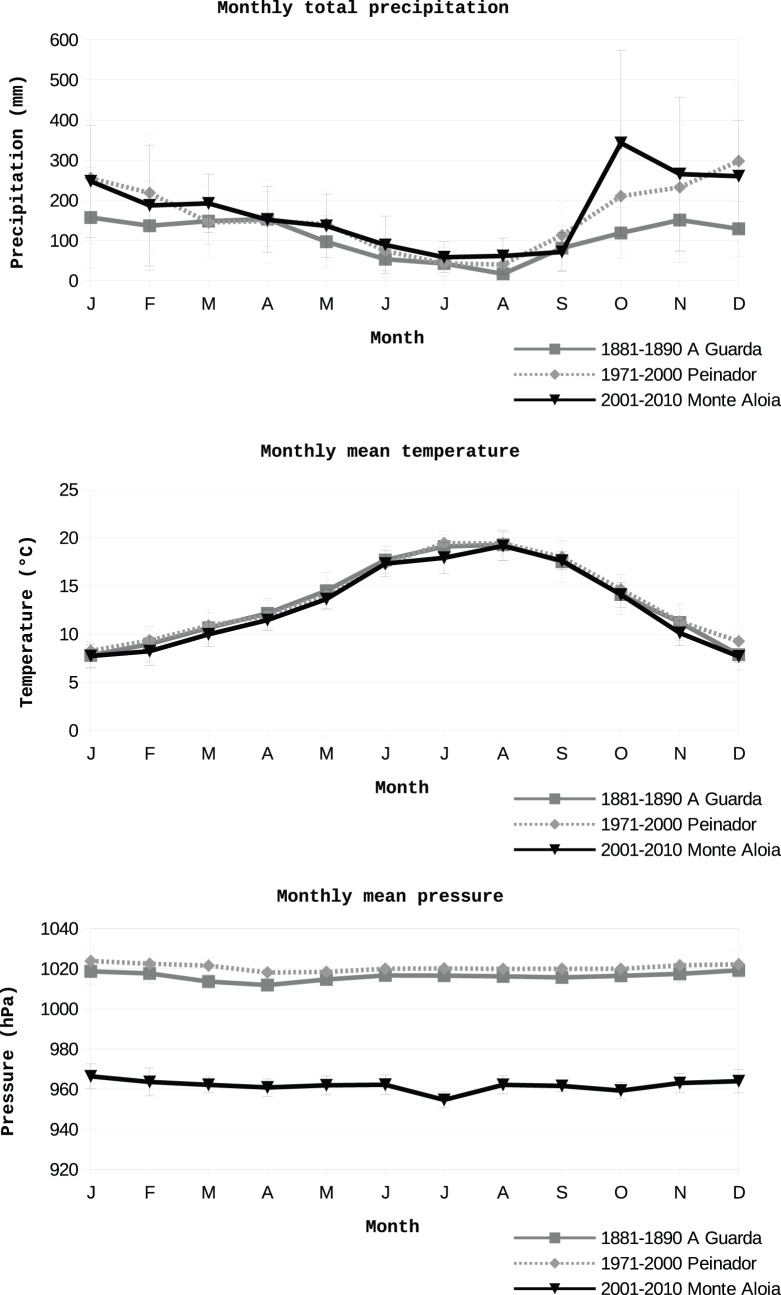
Monthly total precipitation, mean temperature and mean pressure and corresponding standard deviations for the observatory of A Guarda, Monte Aloia and Peinador.

The second part of this notebook contains meteorological data for the period 1894–6, including daily, monthly, seasonal and annual means. The variables recorded are the dominant wind direction, the wind strenght, the cloud coverage and direction, the maximum, minimum and mean temperature, the mean pressure, relative humidity, mean vapour pressure, evaporation, rainfall and also daily subjective records of the observed weather conditions (e.g., “good weather”, “changing weather”, or “bad weather”).

## Results

### Data Series Recovered and Digitized

The data contained in the book are now availabe in OpenDocument format [21], [22], an open and accessible and internationally standard, freely accesible open format available on the internet. The data can be downloaded from: http://ephyslab.uvigo.es/documentos/AGuarda-recovered.ods.

We now list the recovered and digitized data series with the corresponding units where it is of application:

- Notebook I, 1881–1890
  mean monthly pressure (mmHg) and temperature (°*C*), and corresponding maximum and daily oscillations
  mean monthly relative humidity (%), vapour pressure (mmHg) and evaporation (mm)
  total precipitation (mm) and number of rainy days
  number of days with wind from each direction (N, NE, E, SE, S, SW, W, NW)
  number of days with wind according to wind speed (calm, breeze, windy, strong)
  general weather observations (tempest, hail, fog, cloudy, overcast, scatter, clear)
- Notebook II, 1881–1890
  “extratropical cyclone days”
  pressure (mmHg) for extratropical cyclone days
  wind (N, NE, E, SE, S, SW, W, NW) direction for extratropical cyclone days
- Notebook III, 1892–1893
  days of Moon phases
  mean, maximum and minimum daily temperature (°*C*)
  mean, maximum and minimum daily pressure (mmHg)
  temperature in the shade and in the sun (°*C*)
  daily wind speed (between 0 and 3) and direction (N, NE, E, SE, S, SW, W, NW)
  daily relative humidity (%), vapour pressure (mmHg) and evaporation (mm)
  total precipitation (mm)
  cloud cover (between 0 and 2)
  general weather observations (good weather, changing weather, or bad weather)
- **Notebook IV, 1894–1896:** similar to Notebook III, but also includes values of pressure, relative humidity and vapor pressure at 0900 and 1500 UTC.

### Data Series Recovered and Not Digitized

As an additional outcome of our esearch, we also unearthed other documentary sources that could soon contribute to the expansion of the data series thus far recovered and digitized. These are data obtained from Father Orcolaga and the newspaper La Integridad.

**Figure 14:**
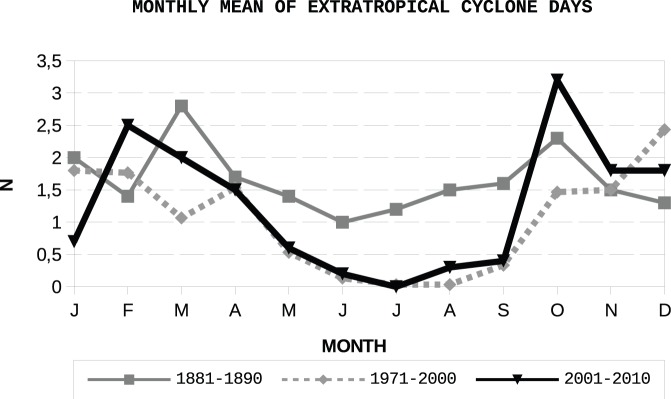
Monthly number of extratropical cyclones for the periods 1881–1890 (A Guarda observatory), 1971–2000 (Peinador station) and 2001–2010 (Monte Aloia station).

**Figure 15:**
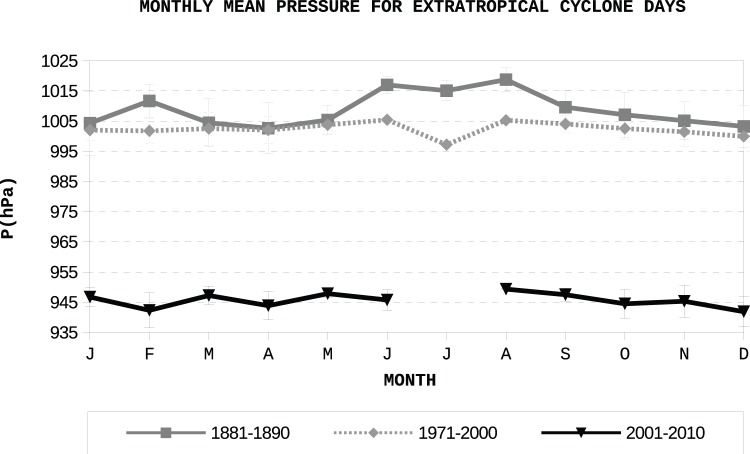
Monthly mean pressures and corresponding standard deviations for extratropical cyclone days for the periods 1881–90 (A Guarda observatory), 1971–2000 (Peinador station) and 2001–2010 (Monte Aloia station).

In the first case, we unexpectedly found a new documentary source at the Observatori de l’Ebre. Father Orcolaga, head of the observatory of Igeldo (Donosti, Spain) received daily reports by telegraph of the data measured at A Guarda, beginning in October, 1904. These reports included data on temperature (in °*C*)(maximum, minimum and mean), vapour pressure, relative humidity, wind speed and direction, cloud type, cover and direction, precipitation, together with annotations describing how to classify the meteorological conditions for each day. The data were measured at 0900 UTC for the period from October 1904 to April 1905, and at 0700 UTC from May 1905 to May 1906. An example of this source of data is given in Table 2 and Figure 10.

Secondly, we recently located a collection of microfilms of local newspaper no longer in circulation called “La Integridad” (Figures 11 and 12) in the library of the Universidad de Santiago de Compostela (Spain). Following a preliminary analysis, it is apparent that daily or weekly meteorological reports obtained from the observatory of A Guarda were occasionally published in this newspaper. However, we have not been able to complete the recovery and digitization of these reports to the date. Table 3 gives a description of this source of data. These reports, known as “Meteorografía”, include mean daily pressure, temperature, daily maximum and minimum temperature, temperature in the sun, maximum and minimum soil temperature at a depth of 30 cm, mean vapour pressure, mean relative humidity, wind speed and direction, subjective weather (cloudy, etc.), rate of soil cooling, precipitation, evaporation and a value of ozone measured. As it can be seen in Figure 12 no units are indicated.

### Comparisons

In order to assess the validity of the data recovered, we performed a comparison using data for the periods 1971–2000 and 2001–2009. We compared the mean monthly temperature, pressure and total precipitation, the number of extratropical cyclones and the monthly mean pressure for extratropical cyclones.

In Figure 13, we compare the monthly total precipitation, monthly mean temperature and pressure and corresponding standard deviation for the periods 1881–1890 (i.e, the recovered data), 1971–2000 (Peinador) and 2001–2010 (the Monte Aloia series). It may be seen that precipitation follows a very similar pattern for the three periods with minimum values in July-August. The values for the two most recent periods closely resemble each other and are generally higher than those for the period of data recovered. The intrannual variability of temperature is extremely similar for both the recovered data and the present data following the typical pattern for extratropical latitudes of the northern hemisphere. The profiles of pressure show the higher corresponding to the hemispheric winter. This is probably a result of the Azores Antyciclone, which is predominant over the region of interest during this season. Its displacement to the Bermudas during the hemispheric summer is probably the cause of the lower mean values of pressure. For 1881–1990 and 1971–2000 the annual minimum pressure occurs in April and for 2001–2010 in July. The absolute values of pressure of A Guarda and Peinador are not comparable with Monte Aloia, but this is simply because of the difference in height between the three stations. The possibility of addressing this by reducing the pressure at Monte Aloia to sea level was not considered feasible, because the the temperature series needed to do this did not have the same temporal homogeneity as the pressure series.

A comparison of the extratropical cyclones shown by the three datasets is not a straighforward matter. From the notebooks, is not clear what Father Merino considers to be an extratropical cyclone. After careful checking of the data, we conclude that it is possible to link the data on extratropical cyclones with a value of pressure. Furthermore, Father Merino seems to state that whenever there is an extratropical cyclone, the wind is at least “fuerte” (strong) or “ventoso” (windy) and sometimes “tempestad” (a tempest). Moreover, in different parts of the notebooks the clear days for A Guarda are linked with values of pressure in excess of 760 mmHg, while 750–760 mmHg correspond to variable weather, 740–750 mmHg corresponds with windy days, 730–740 mmHg corresponds with strong wind days, and values below 730 mmHg correspond with stormy weather. In attending to these criteria and checking the values, we conclude that 755 mmHg represents a better threshold for considering a day to be affected by an extratropical cyclone. We therefore herein consider an extratropical cyclone to be present for those days with values of pressure less than 755 mmHg (aprox. 1006.58 hPa) both for A Guarda and Peinador, where we have data of pressure at sea level. By taking into account the difference in elevation between A Guarda and Monte Aloia, we calculate the corresponding threshold pressure for Monte Aloia using the relationship given by Iribarne and Godson [23]:
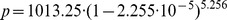



where 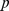 is the pressure in hPa and 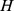 is the altitude in meters. The result is a difference of 56.79 hPa, and we therefore consider extratropical cyclone days for Monte Aloia to be those with pressures less than a threshold value of 949.79 hPa. Figure 14 shows a comparison of these days for the three periods. The three records mostly agree in the shape of the intra-annual variation and in some cases in the actual values. It is important to note how the result values for Peinador mostly agree with those for Monte Aloia and perfectly fit in some cases, which validates the results obtained computing the corresponding sea level pressure. Differences can be because of singular years with an unusual number of extratropical cyclones. This result confirms our correct interpretation of the data contained in the notebooks, and we were therefore able to use them in further comparisons.

Figure 15 shows the intra-annual variation of the mean pressure, using only those days classified as “extratropical cyclone days”. The number of extratropical cyclones used to compute it can be easily followed from Figure 14. The minimum value occurs in 1881–1890 is in April, as for the mean monthly pressure shown in Figure 13. Moreover, for both the recovered series and that obtained from Monte Aloia and Peinador, a steady decrease in pressure may be seen from August to December. As expected, the maximum values of pressure are seen in the hemispheric summer. There were no “extratropical cyclone days” in July at Monte Aloia and just one in Peinador which makes the minimum in pressure less significative than the one for April.

## Discussion

We have herein presented our attempts to recover old instrumental data from documentary sources for the period 1881-96. We have digitized the data series and made them available to the scientific community. We have also compiled an exhaustive set of metadata describing the observatory and its instrumentation. This recovery effort for the northwestern Iberian Peninsula has been enterely worthwhile, as may be seen from our comparisons with more recent data, and wholly relevant for the region of interest, in view of the influence of such phenomena as the North Atlantic Oscillation.

The importance of records of ozone in reports contained in La Integridad can not be overstated; these measurements are not common for the period described here and only a few previous studies have reported similar data for other locations [24]–[26]. It is also most interesting to note the explanation of a procedure for measuring such chemicals as chlorine and ammonia; these reveal the particularly astute observations of Father Merino in his meteorological analysis.

It is unfortunate that the way in which the data were originally reported in the notebooks does not allow more complete climatological comparisons to be made. Nevertheless, it is apparent that they represent a valuable source of information, for example for the study of the timing of extreme meteorological events or for the completion of existing data recovery efforts.
